# Dengue Knowledge and Preventive Practices in Iquitos, Peru

**DOI:** 10.4269/ajtmh.15-0096

**Published:** 2015-12-09

**Authors:** Valerie A. Paz-Soldán, Amy C. Morrison, Jhonny J. Cordova Lopez, Audrey Lenhart, Thomas W. Scott, John P. Elder, Moises Sihuincha, Tadeusz J. Kochel, Eric S. Halsey, Helvio Astete, Philip J. McCall

**Affiliations:** Global Community Health and Behavioral Sciences Department, Tulane University School of Public Health and Tropical Medicine, New Orleans, Louisiana; Facultad de Salud Pública y Administración, Universidad Peruana Cayetano Heredia, Lima, Peru; United States Naval Medical Research Unit No. 6, Iquitos, Peru; Department of Entomology and Nematology, University of California at Davis, Davis, California; Liverpool School of Tropical Medicine, Iquitos, Peru; Vector Biology Department, Liverpool School of Tropical Medicine, Liverpool, United Kingdom; Fogarty International Center, National Institutes of Health, Bethesda, Maryland; Institute for Behavioral and Community Health (IBACH), San Diego State University, San Diego, California; Internal Medicine Department, Hospital Apoyo, Iquitos, Peru; Virology Department, Naval Medical Research Center, Silver Spring, Maryland; Malaria Branch, Centers for Disease Control and Prevention, Atlanta, Georgia

## Abstract

As part of a cluster-randomized trial to evaluate insecticide-treated curtains for dengue prevention in Iquitos, Peru, we surveyed 1,333 study participants to examine knowledge and reported practices associated with dengue and its prevention. Entomological data from 1,133 of these households were linked to the survey. Most participants knew that dengue was transmitted by mosquito bite (85.6%), but only few (18.6%) knew that dengue vectors bite during daytime. Most commonly recognized dengue symptoms were fever (86.6%), headache (76.4%), and muscle/joint pain (67.9%). Most commonly reported correct practices for mosquito control were cleaning homes (61.6%), using insecticide sprays (23%), and avoiding having standing water at home (12.3%). Higher education was associated with higher knowledge about dengue, including transmission and vector control. Higher socioeconomic status was associated with increased reported use of preventive practices requiring money expenditure. We were less likely to find *Aedes aegypti* eggs, larvae, or pupae in households that had < 5-year-old children at home. Although dengue has been transmitted in Iquitos since the 1990s and the Regional Health Authority routinely fumigates households, treats domestic water containers with larvicide, and issues health education messages through mass media, knowledge of dengue transmission and household practices for prevention could be improved.

## Introduction

Dengue viruses (DENVs) are transmitted by the day-biting mosquito *Aedes aegypti* and cause more human morbidity and mortality than any other arthropod-borne virus.^1^,^2^ Annually, there are an estimated 390 million cases of dengue fever worldwide.^3^ Although considerable progress has been made, neither a dengue vaccine nor effective antiviral medication for dengue treatment is currently available.^3^–^5^ As such, vector control remains the primary component of most dengue prevention programs.^3^

DENV transmission has been continuous in Iquitos, Peru, since its reemergence in 1990 after a 30-year absence, with epidemics caused by sequential virus serotype invasions.^6^–^13^ In the city of Iquitos, local health and political authorities conduct routine *Ae. aegypti* control activities including larviciding and health education activities utilizing billboards, radio, and TV messages focusing on preventive vector control activities (container removal and management) and recognition of symptoms, especially early warning signs for severe disease. In response to increases in dengue cases or mosquito indices, emergency measures, indoor space spray applications, and city-wide cleanup campaigns (collection of water-holding container that serve as *Aedes* larval habitats) are instigated.

Despite extensive epidemiologic and entomological studies in Iquitos over the past three decades,^6^–^17^ little has been reported about residents' knowledge of dengue, its vector, and what people do in their homes to prevent infection. Although knowledge does not equate with behavior change, examining what people currently know and do for dengue prevention can help guide future vector control promotion and strategies. With adequate knowledge the community can reduce *Ae. aegypti* production sites in their homes reducing adult indices^18^–^24^ and reduce mortality associated with dengue by recognizing warning signs in ill family members.^18^

As part of a cluster-randomized intervention trial testing the efficacy of insecticide-treated curtains (ITCs) to control DENV transmission, we investigated a wide range of parameters about local knowledge of dengue and its prevention. Data from our knowledge–attitudes–practices (KAP) survey was linked to entomological data from the same households, providing a unique opportunity to analyze reported dengue prevention practices with observed objective outcomes. Herein, we report on 1) knowledge associated with *Ae. aegypti* natural history and control and dengue, 2) individuals' reported perception of risk for dengue disease, 3) reported *Ae. aegypti* control practices in conjunction with objective measures of *Ae. aegypti* population reduction; and 4) sociodemographic and economic factors associated with reported knowledge, attitude, and practice outcomes.

## Methods

### Study site.

The study was carried out in the southern San Juan district (approximate population 102,000)^25^ of Iquitos, which is the largest population center (population approximately 407,000)^25^ in the Peruvian Amazon, located in the northeastern Peru. This district has undergone rapid urbanization and development over the last three decades, and in general, neighborhoods have been more recently incorporated than in other parts of the city; therefore, house construction materials and overall neighborhood infrastructure is of poorer quality or less developed (i.e., dirt versus asphalted streets, underground pipes unavailable).^26^

The cluster-randomized trial was initiated in November 2009, with 10 clusters (each cluster with approximately 90 households distributed between 1 and 3 city blocks) receiving ITC and 10 clusters serving as controls. All clusters were adjacent to one another. In the weeks prior to ITC distribution (November–December 2009), one resident in every intervention and control household (*N* = 1,742) was invited to respond to questions in a baseline KAP survey associated with dengue and *Ae. aegypti* control; 1,333 households participated in the KAP survey (November–December 2009). Baseline serological and entomological samples were collected within weeks of each other (also November–December 2009). Pupal demographic surveys, including immature forms of *Ae. aegypti*, were conducted by a two-person team, and adult mosquitoes of all species, including *Ae. aegypti*, were collected using Prokopack aspirators (Emory University, Atlanta, GA)^27^ and processed as described by Morrison and others.^28^

Participation in the KAP survey was requested from one adult member of each household who was responsible for container management or keeping the household clean and insect free. The KAP survey was made up of close-ended questions developed by a Peruvian social scientist (V. A. Paz-Soldán) utilizing information obtained from ~20 focus groups with Iquitos residents where dengue knowledge and preventive practices were discussed.^29^,^30^ Questions were extensively field tested. The KAP surveyors received training and supervision from the lead social scientist and research team coordinator to ensure consistency. Although this study was part of a larger research project for which entomological and serological data were collected, this manuscript focuses on results from our baseline KAP survey and relevant baseline entomological data linked to outcomes of KAP interest.

### Ethics statement.

Our study received approval from the Institutional Review Boards (IRBs) at the Liverpool School of Tropical Medicine, the Tulane School of Public Health and Tropical Medicine, the London School of Hygiene and Tropical Medicine, the University of California at Davis, and the U.S. Naval Medical Research Center Detachment in Peru. The latter had interinstitutional IRB agreements with the Tulane School of Public Health and Tropical Medicine and the University of California at Davis. The Regional Health Authority (DIRESA), the local branch of the Ministry of Health, also provided approval. The trial was registered with the International Standard Randomized Controlled Trial Number Register: ISRCTN08474420.

### Analysis.

STATA 11.0 software (StataCorp LP, College Station, TX)^31^ was used to calculate medians and frequencies for variables of interest, to conduct χ^2^ tests to examine independence between selected sociodemographic characteristics and variables of interest, and to conduct logistic regressions to calculate odds ratios (ORs) and confidence intervals for the adjusted analyses. Multicollinearity was assessed by testing the correlation between parameters, as well as computing the variance inflation factor to verify the appropriateness of the models. STATA robust cluster commands were used to correct the standard errors estimated for models, using the neighborhood clusters.

Because income does not always capture an individual's wealth or assets adequately, principal component analysis was used to create a socioeconomic status (SES) index variable.^32^ The SES index was created based on the research team's observations of the following variables: housing materials (walls, roof, floor, and window), availability of certain household appliances (refrigerator, TV, DVD, computer, radio, washing machine, motorcycle, and type of stove), number of rooms in the house, and having a phone (landline) and electricity.

### Description of variables.

Most variables used in the analysis were based on participants' responses to survey questions. The research team used information recorded by Regional Health Authority (DIRESA) on the front door of each house as date of the most recent fumigation or larvicide application. The presence of immature forms of *Ae. aegypti* in water containers was obtained from our entomology surveys. Multiple questions were asked to assess knowledge about dengue transmission, prevention, and symptoms. Nine variables were selected as outcome variables for multivariate analyses: two were related to knowledge of dengue transmission, three were related to knowledge of the disease, and four were related to preventive practices (see descriptions in Table 1). Because residents did not differentiate between *Ae. aegypti* and other mosquitoes, participant responses about preventive practices pertain to control of all mosquito species.

## Results

### Study population.

The intervention study included a total of 1,742 lots visited, 1,512 (86.8%) of which were houses. The remaining lots were either vacant houses (188, 10.8%), nonresidential (25, 1.4%; churches, nursery schools, and warehouses), or empty lots (17, 1.0%). Of the residences, 1,345 (89.0%) were enrolled in the ITC study, and of those 1,333 (99.1%) agreed to participate in the KAP survey. Of the remaining houses that did not participate in the study, 82 (4.7%) refused to participate and 85 (4.9%) residents were never found at home despite multiple visits. Hence, our sample consists of 1,333 who completed the KAP survey; and entomological data were available for 1,133 of the households for which we also conducted KAP surveys.

The majority of respondents were women (73.9%). The majority of the interviewees had at least some secondary (56.2%), or postsecondary technical or university education (22.3%; see Table 2). The most frequently cited occupation by respondents was “housewife” (46.0%), followed by unskilled laborer (∼20%; i.e., construction, transportation, and warehouse/shop workers) or “merchants or small business owners” (∼18%; i.e., informal workers at stalls in the market or on their doorstep, with products such as fruits or vegetables).

Within a city block, houses were constructed in rows, sharing walls, which were constructed of wood (58.6%), or bricks and concrete (41.4%), and roofed with corrugated sheet metal (82.5%) or palm leaves (17.5%). Above the shared walls, the majority of houses had high open spaces beneath the roof and open eaves, allowing free indoor movement of mosquitoes. Untreated mosquito bed net use was common but window screens were rare. Nearly one-third (31.8%) of the study population did not have sewage lines or piped water.

### Knowledge of DENV transmission and symptoms.

Although most individuals (85.6%) knew that infected mosquitoes transmit DENV, only 18.6% knew that the dengue vectors bite during the day or early evening (5 am to 8 pm) (Table 3). Most respondents stated that they did not know what time “dengue”-transmitting mosquitoes bite (76.6%). Only 19.4% knew that the vector has white stripes on its legs, and 14.9% knew the name was *Ae. aegypti*. Knowledge about dengue symptoms was higher than about transmission: most interviewees named at least one correct dengue symptom (93.3%); the most symptoms named were fever (86.6%), headache (76.4%), and joint/muscle aches (67.9%).

### Risk perception and preventive practices.

Of the respondents, 65% reported knowing someone who had had dengue—including 20.6% who had a DENV infection themselves—yet only 34.7% considered themselves at high risk of contracting dengue. The two main responses given by respondents on how to prevent dengue (knowledge variable) were to use mosquito bed nets (54.3%) and to clean one's house (46.8%). Other responses included actions to reducing larval development sites by removing (37.1%) or covering (26.4%) water containers. When participants were asked specifically about what they do to reduce mosquito populations, the most common response was cleaning one's home (61.6%). Of the participants, 41% said they applied chemical products such as petroleum, creoline, kerosene, or bleach to their floors to repel mosquitoes. About half of the houses had been fumigated with insecticide (55.4%) or treated with larvicide (58.1%) within 6 months prior to the survey. Overall, both education and SES were positively associated with increased knowledge of DENV transmission, symptoms, and preventive practices in bivariate analyses (see Table 3, columns 3–6).

### Entomological findings.

*Aedes aegypti* eggs, larvae, or pupae were found in 73 houses (6.4%) at baseline. Of these, 58 of the homes (5.1%) had containers considered useless by residents. The most common adult mosquito species collected were *Culex* spp. (81.5%), followed by *Ae. aegypti* (17.6%). At least one adult *Ae. aegypti* was collected in 37.4% of the homes and at least one adult female *Ae. aegypti* was collected in 26.6% of the homes. Adult mosquito samples ranged from 0 to 294, with a median of 3. The mean number of male mosquitoes per home (4.15) collected was slightly higher than females (3.32).

### Examining socioeconomic and demographic factors associated with DENV knowledge and preventive practices.

In adjusted models, we found that more education (secondary or university studies versus having 7 years or less of school), knowing someone who had had dengue (versus not), and being female were all significantly, positively associated with the two selected knowledge variables about dengue transmission (see Table 4). Hence, the odds of knowing that DENV is transmitted by infected *Ae. aegypti* (OR = 5.0, *P* < 0.01) or that this mosquito bites during the day/early evening (OR = 3.1, *P* < 0.01) were higher in the highest educational group (technical or university studies) compared with those with fewer than 7 years of education.

Being in the higher educational groups, being in the highest SES category, knowing someone who had had dengue, and being female were also significantly, positively associated with knowing three or more dengue symptoms, one or more ways to prevent DENV infection, and the utility of paracetamol for relief from dengue symptoms (see Table 5). The odds of knowing at least three dengue symptoms and of paracetamol for dengue relief were highest among those who knew someone who has had dengue (OR = 2.7, *P* < 0.01; OR = 2.8, *P* < 0.01, respectively) compared with those who reported not knowing anyone who had dengue. The odds of women knowing at least three dengue symptoms (OR = 2.3, *P* < 0.01) and that one should take paracetamol for relief (OR = 2.6, *P* < 0.01) were also significantly higher than for men.

Respondents in the highest and middle SES groups were 3.8 (*P* < 0.01) and 1.8 times (*P* < 0.01), respectively, more likely to report purchasing an effective vector control product (insect spray) than those in the lowest SES group (Table 6). Similarly, larvicide use (Table 1) showed a similar trend with greater observed use among those in the highest (OR = 2.6, *P* < 0.01) and middle (OR = 1.8, *P* < 0.01) SES groups compared with the lowest SES group.

Education was the only factor significantly associated with reported activities to reduce larval development sites—those in the highest educational group were 2.4 times more likely to report taking actions to reduce breeding sites than those with fewer than 7 years of education (*P* < 0.01). Entomological surveys corroborated participant responses; of 73 houses with *Ae. aegypti* of any developmental stage, only three (4.2%) were in households that reported practices to remove vector breeding sites, and no unused containers with standing water were observed in these houses.

Examining associations with a verified entomological outcome (absence of *Ae. aegypti* of any developmental stage in that home) that could be attributed to a reported practice (i.e., reduction of development sites), households where respondents had children under 5 years of age were significantly less likely to have *Ae. aegypti* present (OR = 1.95, *P* < 0.01). Examining the association between the sociodemographic and economic variables and presence of at least one adult *Ae. aegypti* in the home or at least one adult female *Ae. aegypti* showed a positive association between older (over 44 years of age) respondents for both outcomes (OR = 1.5, *P* < 0.01 for both). We also found a positive association between those with highest levels of education and presence of at least one adult *Ae. aegypti* (OR = 1.4, *P* < 0.05).

## Discussion

Higher education levels were associated with better knowledge about dengue, preventive practices, and with reported attempts to reduce mosquito production sites. Higher SES was associated with higher reported use of protective measures that required spending money, for example, insect spray. Knowledge about dengue and its symptoms was higher among women than men, perhaps because they have more of a role in caring for ill family members. Not surprisingly, higher knowledge about dengue and its symptoms was associated knowing someone who had had dengue in the past.

In contrast, the knowledge about *Ae. aegypti*'s daytime biting behavior and the ability to distinguish it from other mosquito species were poor. When interviewing participants about dengue prevention practices, we asked about mosquito control practices generally, rather than *Ae. aegypti*, because most interviewees did not differentiate between mosquito species. From a programmatic standpoint, health education messages could be designed to improve people's knowledge of DENV transmission, emphasizing on the different kinds of mosquitoes and that prevention may not work on all species, to improve acceptability of dengue-specific products and practices (i.e., indoor emergency space sprays). For example, findings from a qualitative study in this community revealed that interventions targeting *Ae. aegypti* were perceived as ineffective if other nuisance mosquitoes were not also affected (unpublished data), potentially leading to the perception of product inadequacy or even failure, and potentially eventual abandonment.

While some knowledge of dengue prevention practices was incorrect, notably, the use of bed nets for protection against diurnally active *Ae. aegypti* (although this is not one of the practices recommended for dengue control in Iquitos, recent literature suggests bed nets could reduce *Ae. aegypti* infestations in houses^33^), many correctly identified proper methods of reducing mosquito production and were consistent with well-publicized vector control messages, that is, house cleaning, removal and management of water containers. Improving people's knowledge about dengue transmission may lead to increased understanding and reinforcement of why certain practices are more effective for dengue prevention than others (i.e., reducing larval development sites in the home) and why others may not reduce dengue, which in turn may help people make better choices regarding which preventive practices to implement.

In bivariate analyses, higher education and SES levels were strongly associated with greater knowledge of DENV transmission and prevention and with active mosquito control practices. When these variables and others were included in the multivariate analysis, we observed that persons with higher education were more likely to know about DENV transmission and report preventive actions to reduce mosquitoes that were of no cost. Only those with higher SES were more likely to report taking action with costly products, such as insecticide sprays or other chemical options. It is possible that the middle to high SES group was more likely than the lower SES group to use relatively expensive insecticide aerosol sprays, which can reduce DENV transmission,^34^ because they could afford them. For this reason, under emergency conditions, the feasibility of subsidizing costs of effective consumer product interventions merits further examination as an alternative rapid response to city-wide adulticide spray campaigns led by the regional health authority that are often delayed due to government budget constraints and implementation logistics.

We detected a positive significant association between acceptance of larviciding and SES (but not with education levels), but unlike the case with insecticide sprays, there are no costs associated with larviciding. This result was puzzling; larviciding, as well as fumigation, is carried out by the regional health authority and neither education level nor SES was associated with compliance in fumigation campaigns (data not shown). One possibility is that those with higher SES were more likely to have larger water storage containers, cisterns, and even swimming pools that would require larvicides. Another possibility might be related to timing: fumigation often occurs around 5 am or 5 pm when most people, regardless of SES, are home, while larviciding occurs throughout the day. Potentially, people in higher SES groups could be more likely to have someone at home at all times who can let the regional health authority team in. Alternatively, fumigation could have simply been more acceptable than larviciding, or the more frequent application of larvicides could have led to decreased confidence in this intervention. More study is required to understand why compliance with larviciding, a relatively cost-effective way to reduce mosquito production, was greater in the higher SES groups.

The presence of *Ae. aegypti* eggs, larvae, or pupae was less likely in houses where there was a child under 5 years of age. The practices of keeping yards and houses free of standing water, trash containers, and other potential mosquito breeding sites requires time and effort. Individuals with young children (< 5 years of age) may have been more motivated to keep their homes free of mosquitoes to protect their children. It is also possible that mothers with young children were more likely to spend more time at home, with more opportunities to clean the home environment.

We identified a strong positive association between reported prevention behaviors to reduce larval development sites and a higher level of education. Though not statistically significant, those with higher education were more likely to have *Ae. aegypti* eggs, larvae, or pupae in their homes, contradicting their reported mosquito control practices. Note that the presence of *Ae. aegypti* eggs, larvae, or pupae in the household was the closest indicator of behavior to reduce mosquito development sites, rather than directly observed evidence of those practices. Doing this, however, would be limited by people changing their behavior in response to the observer and the ability to validate that the practice was sustained overtime. Alternatively, it is possible that those with higher education did not actually reduce breeding sites around their homes any more than others, but they reported this due to social desirability bias, for example, the knowledge that this is considered a desirable behavior by others. It is also possible that the houses of people with higher education were larger or constructed differently, making container management more difficult—although we would have expected to see this effect more strongly among those in a higher SES versus those with highest education. Though it was not a goal of this study to evaluate health education, Iquitos residents are exposed to dengue health education messages primarily focused on covering, cleaning, or removing containers that accumulate water. Those with higher education levels may be more likely to understand the messages (whether they carry them out or not), or may feel more confident in their ability to carry out the recommended practices. One programmatic implication from our findings is that health education messages need to be field tested with diverse audiences to ensure the message is clear and understandable to those with less education.

Knowledge about a subject does not always translate into behavior change—for example, despite well-known benefits from exercise, many do not exercise. In locations like Iquitos, although you might take preventive measures in your own home, residents cannot control practices of their neighbors or their exposure outside their home. Some individuals may feel unmotivated to do anything simply because dengue may be perceived as “inevitable” to them regardless of what they do. That said, at a population scale, we would expect that some individuals would be motivated to reduce their or their family's risk if they know what to do. Beyond traditional health promotion activities, learning from others around one (social learning) or feeling normative pressure from neighbors or friends to implement preventive strategies at home should lead to others carrying out these practices at home. However, for any of this to occur, there needs to be a certain amount of awareness and education in the community about dengue, how it is transmitted, and what one can do to reduce transmission. This health education can be promoted via various methods: health education campaigns, word of mouth (sometimes triggered by conversations about a campaign), or health workers. In a future study, we plan to examine whether our dengue research team's long-term presence in certain communities in Iquitos has resulted in higher dengue knowledge and increased preventive practices in these communities compared with others–we hypothesize this may have been an unintended, but positive, effect of many years of presence in some communities.

Despite exposure to dengue-related information over many years, Iquitos residents' knowledge about DENV transmission and its prevention can be improved. Programmatic implications include increasing health education about DENV transmission and ensuring health education materials are developed for people with a range of education backgrounds. More research is necessary to understand larviciding compliance, a program provided at no cost by vector control authorities, to ensure all households can benefit from it. A cost-effectiveness analysis would also help to determine whether subsidizing commercially available products with proven efficacy for adult mosquito control could increase preventive practices. Improvements in dengue knowledge and preventive practices in Iquitos are needed to enhance the success of community-based aspects of dengue intervention methods and strategies.

## Figures and Tables

**Table 1:** Description of outcome variables used in multivariate analyses

Topics of interest	Definition of outcome variables used in the analysis
Knowledge of dengue transmission	Respondent stated that dengue is transmitted by the bite of an infected mosquito
	Respondent stated that mosquito vector that transmits dengue bites during day hours, defined this way if the respondent mentioned any hours between 5 am and 8 pm
Knowledge of the disease	Respondent was able to name at least three correct (and typical) symptoms in dengue patients
	Respondent answered one should take paracetamol (acetaminophen) for dengue symptom relief
	Respondent was able to name at least one appropriate and correct household practice that could prevent dengue
Household practices to reduce mosquitoes at home	Respondent reports use of insect spray (representing an effective mosquito control product that is purchased by the respondent)
	Respondent allows use of larvicide (applied by vector control personnel at no cost to the resident). Use of larvicide is confirmed by research team who check for sticker with date placed on respondent's door by vector control personnel
	Respondent reports any appropriate physical intervention to reduce mosquitoes (including removing useless containers and items that may collect standing water and covering water containers)
	Entomology team does not find any *Aedes aegypti* eggs, larvae, and/or pupae in any water container within the house

**Table 2:** Sociodemographic characteristics of sample (*N* = 1,333)

Characteristics	Frequency
	% (*n*)
Age	39 (median), 16–88 (range)
Sex	
Female	73.9 (985)
Male	26.1 (348)
Education*	
< 7 years	21.5 (287)
7–11 years	56.2 (749)
> 11 years	22.3 (297)
Occupation	
Housewife	46.0 (613)
Merchant/small businessmen	17.6 (235)
Unskilled labor	20.6 (274)
Skilled, independent labor	6.2 (82)
Health/education professionals	5.8 (77)
Unemployed/retired	1.8 (24)
Student	2.1 (28)
Household information	
Number of people living in home	5 (median), 1–17 (range)
Have children < 5 years old at home	39.5 (526)
Pregnant woman/women living at home	7.1 (94)

* In Peru, elementary school consists of an initial 6 years, and high school is of further 5 years. Postsecondary education may consist of university or technical programs.

**Table 3:** KAP associated with dengue and frequencies by education and socioeconomic status

	Total (*N* = 1,333)	Education < 7 years (*N* = 287)	Education > 11 years (*N* = 298)	Lowest SES (*N* = 348)	Highest SES (*N* = 450)
	% (*n*)	% (*n*)	% (*n*)	% (*n*)	% (*n*)
Knowledge on dengue transmission					
Dengue is transmitted through mosquito bite	85.6 (1,141)	75.6 (217)**	93.6 (279)**	82.2 (286)*	88.4 (398)*
*Aedes aegypti* transmits dengue	14.9 (198)	1.7 (5)**	34.2 (102)**	7.8 (27)**	22.4 (101)**
“Dengue” mosquito usually bites during					
Day/evening	18.6 (248)	12.2 (35)**	29.2 (87)**	15.8 (55)	21.8 (98)*
Does not know	76.6 (1,021)	81.5 (234)*	67.1 (200)**	78.7 (274)	73.8 (332)
Knowledge about dengue illness					
Knows following dengue symptoms					
Fever	86.6 (1,154)	79.4 (228)**	90.6 (270)	83.9 (292)	90.4 (407)**
Headache	76.4 (1,019)	72.8 (209)	75.2 (224)	75.3 (262)	79.8 (359)*
Muscle/joint/body pain	67.9 (905)	67.3 (193)	69.8 (208)	64.4 (224)	71.1 (320)
Nausea/vomiting	25.1 (334)	24.0 (69)	26.9 (80)	23.6 (82)	26.0 (117)
Rash	8.1 (108)	4.5 (13)*	9.4 (28)	6.6 (23)	8.4 (38)
Loss of appetite	5.3 (70)	4.9 (14)	9.7 (29)**	3.2 (11)*	8.0 (36)**
Bleeding from gums, nose, or mouth	5.1 (68)	3.1 (9)	4.7 (14)	3.5 (12)	5.6 (25)
Eye pain	4.7 (62)	3.5 (10)	6.4 (19)	4.3 (15)	5.1 (23)
Knows three or more correct symptoms	76.1 (1,015)	70.0 (201)**	77.2 (230)	71.6 (249)*	81.3 (366)**
Knowledge on preventive practices					
One can prevent dengue by					
Using mosquito nets	54.3 (724)	48.8 (140)*	58.4 (174)	51.4 (179)	53.6 (241)
Cleaning one's house	46.8 (624)	43.6 (125)	48.0 (143)	42.8 (149)	51.8 (233)**
Removing useless water-collecting containers	37.1 (495)	32.1 (92) *	45.0 (134)**	33.1 (115)	44.0 (198)**
Covering water containers	26.4 (352)	20.6 (59)*	26.5 (79)	25.9 (90)	28.0 (126)
Fumigating one's house	17.7 (236)	12.2 (35)**	23.5 (70)**	14.7 (51)	20.9 (94)*
Using various chemical products	13.5 (180)	10.1 (29)	14.4 (43)	9.2 (32)**	17.8 (80)**
Reducing standing water in house	4.5 (60)	2.4 (7)	6.4 (19)	2.6 (9)*	4.4 (20)
Treating the water (with bleach, etc.)	3.7 (49)	3.1 (9)	3.4 (10)	3.5 (12)	4.2 (19)
Using insect repellent	2.0 (26)	1.4 (4)	4.0 (12)**	1.2 (4)	2.7 (12)
Perception of risk					
Know someone who has had dengue	65.0 (866)	59.9 (172)*	75.2 (224)**	62.1 (216)	67.1 (302)
Practices to reduce mosquitoes					
Cleans the house	61.6 (821)	61.0 (175)	62.4 (186)	57.5 (200)	67.3 (303)**
Uses petroleum, creoline, kerosene, or bleach	41.0 (547)	42.2 (121)	33.9 (101)**	35.6 (124)*	42.7 (192)
Uses insecticide spray	23.0 (307)	17.1 (49)**	30.5 (91)**	11.8 (41)**	35.3 (159)**
Avoids having standing water in house	12.3 (164)	9.8 (28)	18.5 (55)**	12.0 (42)	12.9 (58)
Uses insecticide coils	10.8 (144)	9.8 (28)	11.4 (34)	10.6 (37)	13.3 (60)*
Burns leaves and other items to smoke out mosquitoes	6.7 (89)	9.8 (28)*	6.4 (19)	8.6 (30)	5.6 (25)
Takes out trash	4.2 (56)	3.1 (9)	4.7 (14)	4.3 (15)	3.8 (17)
Carries out physical intervention at home†	66.5 (886)	64.5 (185)	71.5 (213)*	61.8 (215)*	72.4 (326)**
Reduces breeding sites through various activities	14.8 (197)	11.9 (34)	21.5 (64)**	14.9 (52)	15.1 (68)
Outside interventions for mosquito control					
Home fumigated in last 6 months	55.4 (739)	53.3 (153)	53.7 (160)	54.0 (188)	55.6 (250)
Larvicide used in home in last 6 months	58.1 (775)	56.5 (162)	64.1 (191)*	44.0 (153)**	68.7 (309)**
Fumigation was conducted by					
Regional health authority or municipality	84.3 (1,124)	87.5 (251)	82.6 (246)	84.2 (293)	84.0 (378)
Home owner	4.1 (54)	2.8 (8)	6.7 (20)**	0.6 (2)**	8.2 (37)**
Entomological variables					
Useless containers found in home	5.1 (58)	4.7 (12)	4.7 (11)	5.3 (16)	4.3 (16)
Larvae, pupae, or eggs are found in home	6.4 (73)	7.0 (18)	8.2 (19)	7.3 (22)	5.6 (21)

KAP = knowledge, attitudes, and practices; SES = socioeconomic status.

* *P* < 0.05; **P < 0.01.

† Nonchemical and non-purchasable.

**Table 4:** Multivariate analysis of sociodemographic and economic factors associated with knowledge of dengue transmission, reporting ORs and 95% CIs (*N* = 1,333)

	Dengue transmitted by bite of infected mosquito	Mosquito that transmits dengue bites during day/early evening
	OR (95% CI)	OR (95% CI)
Housewife	1.21	0.93
	(0.769, 1.889)	(0.637, 1.371)
< 30 years old	0.82	0.97
	(0.544, 1.251)	(0.645, 1.456)
> 44 years old	0.88	1.42*
	(0.585, 1.321)	(1.022, 1.969)
Female	1.35	1.33
	(0.862, 2.120)	(0.903, 1.946)
7–11 years of education†	2.05**	1.63**
	(1.488, 2.812)	(1.129, 2.348)
≥ 11 years of education†	4.95**	3.08**
	(2.593, 9.455)	(1.928, 4.923)
Middle SES‡	1.08	0.94
	(0.735, 1.577)	(0.635, 1.402)
Highest SES	1.21	1.02
	(0.821, 1.783)	(0.732, 1.431)
Know someone who has had dengue	1.88**	2.23**
	(1.444, 2.460)	(1.494, 3.315)
Child < 5 years living in home	1.26	0.80
	(0.948, 1.681)	(0.591, 1.090)

CI = confidence interval; OR = odds ratio; SES = socioeconomic status.

* *P* < 0.05; **P < 0.01.

† Reference group are those with less than 7 years of education.

‡ Reference group are those in the lowest SES group.

**Table 5:** Multivariate analysis of sociodemographic and economic factors associated with knowledge of dengue symptoms, treatment, and prevention (*N* = 1,333)

Sociodemographic characteristics	Knows three or more dengue symptoms	Knows to take paracetamol for dengue	Knows at least one way to protect from dengue
	OR (95% CI)	OR (95% CI)	OR (95% CI)
Housewife	1.47*	1.46*	1.12
	(1.056, 2.040)	(1.082, 1.961)	(0.821, 1.538)
< 30 years old	0.66	0.77*	1.17
	(0.399, 1.077)	(0.589, 0.997)	(0.841, 1.637)
> 44 years old	0.77	0.81	1.04
	(0.585, 1.024)	(0.615, 1.060)	(0.791, 1.375)
Female	2.32**	2.70**	1.21
	(1.831, 2.937)	(2.094, 3.493)	(0.897, 1.634)
7–11 years of education†	1.62**	1.14	1.74**
	(1.167, 2.246)	(0.782, 1.669)	(1.329, 2.284)
≥ 11 years of education†	1.64*	1.64	2.69**
	(1.029, 2.599)	(0.977, 2.756)	(1.534, 4.701)
Middle SES‡	1.08	1.27	1.01
	(0.762, 1.517)	(0.916, 1.748)	(0.754, 1.346)
Highest SES	1.66**	1.54*	1.69*
	(1.155, 2.373)	(1.032, 2.306)	(1.054, 2.719)
Knows someone who has had dengue	2.75**	2.88**	1.23
	(2.176, 3.465)	(2.301, 3.613)	(0.944, 1.591)
Child < 5 years of age in home	1.04	1.16	1.04
	(0.786, 1.365)	(0.883, 1.514)	(0.783, 1.386)

CI = confidence interval; OR = odds ratio; SES = socioeconomic status.

* *P* < 0.05; **P < 0.01.

† Reference group are those with less than 7 years of education.

‡ Reference group are those in the lowest SES group.

**Table 6:** Multivariate analysis of sociodemographic and economic factors associated with reported and observed household practices to prevent dengue, as well as the absence of immature forms of *Aedes aegypti* (*N* = 1,333)

Sociodemographic characteristics	Uses insect spray†	Larvicide used in house in last 6 months (verified) (*N* = 1,133)	Reported reduction of breeding sites	Absence of *Ae. aegypti* eggs, larvae and/or pupae (*N* = 1,133)
	OR (95% CI)	OR (95% CI)	OR (95% CI)	OR (95% CI)
Housewife	0.89	1.26	1.09	1.62
	(0.631, 1.256)	(0.934, 1.688)	(0.745, 1.590)	(0.968, 2.714)
< 30 years old	1.47*	0.81	1.08	1.05
	(1.008, 2.135)	(0.575, 1.130)	(0.707, 1.652)	(0.508, 2.180)
> 44 years old	1.07	1.31	1.48	1.38
	(0.792, 1.436)	(0.972, 1.772)	(0.917, 2.379)	(0.797, 2.385)
Female	0.90	1.04	0.93	1.08
	(0.653, 1.228)	(0.775, 1.388)	(0.603, 1.421)	(0.560, 2.094)
7–11 years of education	1.16	1.05	1.29	1.35
	(0.820, 1.630)	(0.810, 1.355)	(0.851, 1.970)	(0.810, 2.237)
≥ 11 years of education	1.23	1.27	2.35**	1.02
	(0.830, 1.829)	(0.973, 1.650)	(1.459, 3.790)	(0.453, 2.292)
Middle SES	1.80**	1.76**	0.84	1.17
	(1.225, 2.644)	(1.288, 2.416)	(0.514, 1.376)	(0.653, 2.092)
Highest SES	3.83**	2.59**	0.75	1.39
	(2.517, 5.826)	(1.736, 3.870)	(0.443, 1.283)	(0.690, 2.785)
Knows someone who has had dengue	1.11	1.12	1.32	1.42
	(0.785, 1.568)	(0.830, 1.498)	(0.912, 1.904)	(0.835, 2.427)
Child < 5 years living in home	0.67*	1.04	0.84	1.95**
	(0.468, 0.958)	(0.831, 1.303)	(0.611, 1.155)	(1.313, 2.907)

CI = confidence interval; OR = odds ratio; SES = socioeconomic status.

Most preventive practices are based on respondents' reported practices and labeled as such (i.e., “reported” practice); larviciding and absence of *Ae. aegypti* eggs, larvae, and pupae were verified by the research team, and only in 1,133 of the households.

* *P* < 0.05; **P < 0.01.

† Results for reported use of any chemical product, whether truly preventative or not, were similar to reported use of insect spray.
